# Evolution of Diagnostic Tests for Chronic Wasting Disease, a Naturally Occurring Prion Disease of Cervids

**DOI:** 10.3390/pathogens6030035

**Published:** 2017-08-05

**Authors:** Nicholas J. Haley, Jürgen A. Richt

**Affiliations:** 1 Department of Microbiology and Immunology, Arizona College of Osteopathic Medicine, Midwestern University, Glendale, AZ 85308, USA; 2 College of Veterinary Medicine, Kansas State University (KSU), Manhattan, KS 66506, USA; jricht@vet.k-state.edu

**Keywords:** prion, cervids, PMCA, RT-QuIC, diagnosis

## Abstract

Since chronic wasting disease (CWD) was first identified nearly 50 years ago in a captive mule deer herd in the Rocky Mountains of the United States, it has slowly spread across North America through the natural and anthropogenic movement of cervids and their carcasses. As the endemic areas have expanded, so has the need for rapid, sensitive, and cost effective diagnostic tests—especially those which take advantage of samples collected antemortem. Over the past two decades, strategies have evolved from the recognition of microscopic spongiform pathology and associated immunohistochemical staining of the misfolded prion protein to enzyme-linked immunoassays capable of detecting the abnormal prion conformer in postmortem samples. In a history that parallels the diagnosis of more conventional infectious agents, both qualitative and real-time amplification assays have recently been developed to detect minute quantities of misfolded prions in a range of biological and environmental samples. With these more sensitive and semi-quantitative approaches has come a greater understanding of the pathogenesis and epidemiology of this disease in the native host. Because the molecular pathogenesis of prion protein misfolding is broadly analogous to the misfolding of other pathogenic proteins, including Aβ and α-synuclein, efforts are currently underway to apply these in vitro amplification techniques towards the diagnosis of Alzheimer’s disease, Parkinson’s disease, and other proteinopathies. Chronic wasting disease—once a rare disease of Colorado mule deer—now represents one of the most prevalent prion diseases, and should serve as a model for the continued development and implementation of novel diagnostic strategies for protein misfolding disorders in the natural host.

## 1. Background and Introduction

Chronic wasting disease (CWD) is a naturally occurring transmissible spongiform encephalopathy (TSE) known to affect a range of cervid species, including white-tailed and mule deer (*Odocoileus virginianus* and *Odocoileus hemionus*), North American elk (wapiti, *Cervus elaphus elaphus*), moose (*Alces alces*), and reindeer (*Rangifer tarandus*) [1,2,3]. Since its initial discovery nearly 50 years ago in Northern Colorado and Southern Wyoming, the disease has been reported in 22 additional states, 2 Canadian provinces, South Korea, and very recently in Norway (see https://www.nwhc.usgs.gov/disease_information/chronic_wasting_disease/ for a current map of the geographic extent of CWD in North America). As with other TSEs, including scrapie of sheep, bovine spongiform encephalopathy (BSE), and human variant and sporadic Creutzfeldt-Jakob disease (CJD), CWD is characterized by central nervous system pathology mediated by an abnormally folded isoform of the normal cellular prion protein (PrP^res^ when referring to the misfolded variant or PrP^Sc^ when referring to the infectious isoform specifically, and PrP^C^, respectively). The primary structure of PrP^C^, dictated by the host’s prion protein gene (*PRNP*), plays a vital role in intra- and inter-species susceptibility, reducing susceptibility in animals with specific alleles and serving as the basis for the “species barrier”, limiting the disease almost exclusively to cervids [4,5,6,7,8,9]. The molecular pathogenesis of prion diseases like CWD shares many common traits with other protein misfolding disorders, including Alzheimer’s disease and Parkinson’s disease, and while most prion diseases are decreasing or stable in prevalence, the ever-expanding range of CWD makes it a tempting model system for the broad development of novel diagnostic approaches for these proteinopathies.

In its present range, CWD has been found among both farmed and free-ranging cervids [2]. Although most evidence is anecdotal, both farmed and free-ranging animals have played a role in the progressive spread of the disease across North America and to South Korea [10,11,12]. The recent discovery of CWD in Norway is perplexing, with wildlife managers scrambling to determine not only the extent of infection, but also its source—whether arising in situ or imported in some form from North America [3,13]. While the natural or anthropogenic movement of animals may play the most prominent role in the spread of CWD, the movement of animal carcasses has likely also been involved in dissemination [14,15]; the role of animal byproducts and bodily fluids is less clear, although tissues and bodily fluids including deboned muscle [16] and fat [17], antler velvet [18], saliva [19,20], feces [21], and urine [19] have proven infectious under experimental conditions.

Making matters more difficult is the protracted nature of the disease—whereby several years may pass between preliminary exposure and the onset of clinical symptoms. Not unlike the pathogenesis of rabies infection in animals, the infectious PrP^Sc^ protein must make its way from the periphery, most likely following oral exposure, to the central nervous system. The spread to peripheral excretory tissues, either concurrent with or pursuant to CNS infection, permits the shedding of de novo misfolded PrP^Sc^ into the environment, perpetuating the transmission cycle. Most research has shown that the appearance of the misfolded PrP^Sc^ isoform in peripheral tissues and the onset of shedding may take place months or perhaps years before the appearance of clinical signs. Preclinical peripheral accumulation of prions and shedding in bodily fluids greatly contribute to the imperceptible movement of disease to CWD-free areas via infected animals and those byproducts described above.

As a result of disease expansion and the risks that the movement of clinical or pre-clinical animals, their carcasses, and byproducts may play in transmission, some urgency has been placed on the development of diagnostic approaches which are rapid, sensitive, cost effective, and can make use of samples collected either postmortem or antemortem. Paralleling the history of more conventional infectious agents, the evolution of prion diagnostic strategies has progressed first from the identification of characteristic microscopic pathologic changes [22], to antibody-antigen dependent detection systems [23,24,25,26], and eventually to the advent of techniques for the isolation [27] and amplification [28,29,30,31,32] of the building blocks of stored biological information—in the case of TSEs, the very structure of the prion protein itself.

Building on these approaches, new strategies are being developed to allow for the quantification of prion burden in a tissue, body fluid, or environmental sample. Perhaps a loftier goal, the development of in vitro techniques which may allow for strain discrimination would be tremendously helpful in identifying the source of recent or historic introductions of the disease across North American and now Scandinavia. As these approaches are implemented and refined for the detection of CWD, they will likewise lead to suitable diagnostic tests to meet objectives for the diagnosis of prions and other protein misfolding disorders.

## 2. The History of CWD Diagnostics

Roughly 12 years passed between the early clinical recognition of chronic wasting disease in the 1960s and its definitive grouping within the rapidly growing category of transmissible spongiform encephalopathies soon to be recognized globally as “prion” diseases [1]. The original clinical descriptions of CWD in mule deer are still appropriate today—a syndrome of slowly progressive neurologic dysfunction, behavioral changes, polyuria, polydipsia and hypersalivation, dysphagia and occasional aspiration pneumonia, and ultimately, death [2,33,34]. Like many other TSEs, postmortem diagnoses were based primarily on characteristic neurohistopathologic changes in the gray matter at all levels of the CNS—the spinal cord, mesencephalon, diencephalon, and both cerebellar and cerebral cortices. At the heart of the clinical signs, pathognomonic central nervous system lesions consisted of microcavitation of the neuropil, intracytoplasmic vacuolization, astrocytic hypertrophy and hyperplasia, and neuronal degeneration. Cached amongst these CNS lesions: amyloid plaques, best observed with Congo red or Bodian silver staining. Although the nature and origin of these plaques were unknown at the time, they were a consistent finding across TSEs of both animals and man.

**Figure 1:**
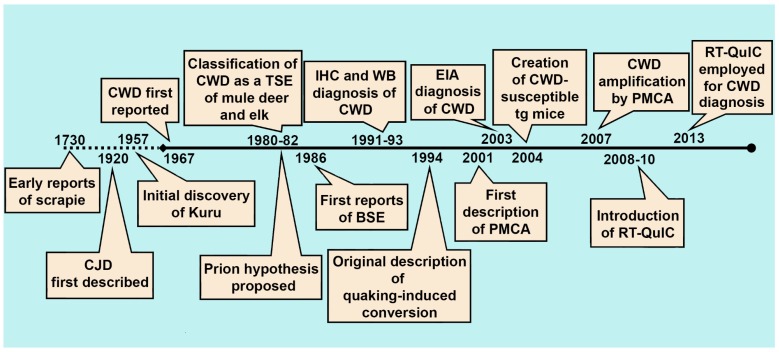
History of diagnostic developments for chronic wasting disease (CWD) and other transmissible spongiform encephalopathies (TSEs). CJD: Creutzfeldt-Jakob Disease; BSE: bovine spongiform encephalopathy; IHC: immunohistochemistry; WB: western blotting; EIA: enzyme immunoassay; PMCA: protein misfolding cyclic amplification; RT-QuIC: real time quaking-induced conversion.

With the definitive identification of the agent responsible for prion diseases, an abnormally folded and hardy conformer of the cellular prion protein [35,36], very specific immunoassays would be developed that could be used on a range of platforms, including fresh and fixed tissues. In cases where these immunoassays were not sensitive enough, bioassay in susceptible hosts—occasionally requiring serial passage—became the *de facto* testing method for infectivity. Each of these has served its respective fields—diagnostic medicine and research, for more than 20 years (Figure 1).

### 2.1. Immunohistochemistry, Western Blotting, and Enzyme Immunoassay

**Figure 2:**
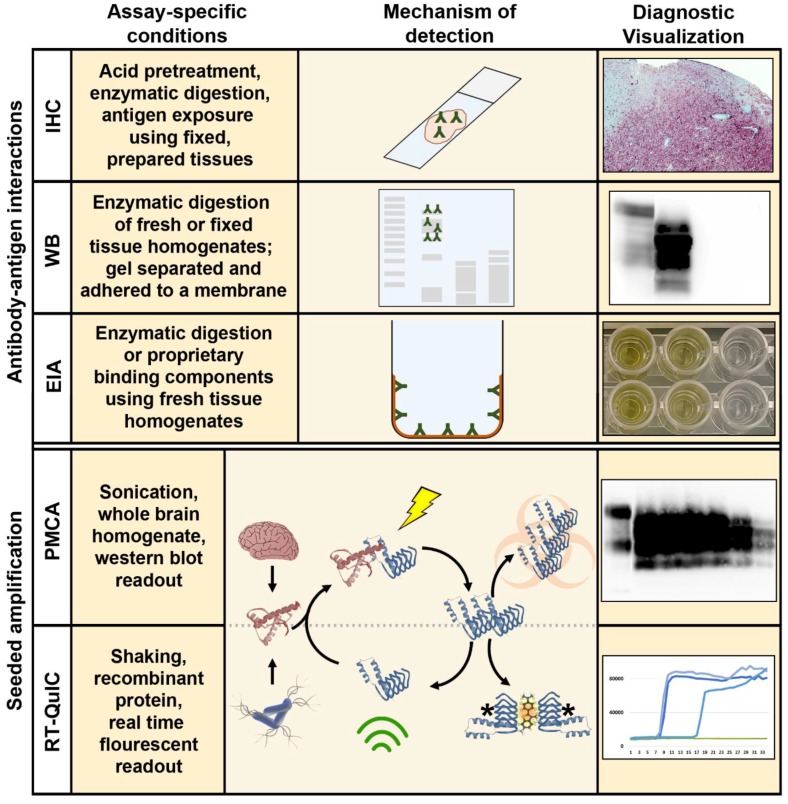
Summary of conventional CWD diagnostic strategies and seeded amplification methods for amplifying CWD prions in vitro. Distinguishing conditions for each assay, as well illustrative mechanisms of detection and representative diagnostic results are presented. IHC: immunohistochemistry; WB: western blotting; EIA: enzyme immunoassay; PMCA: protein misfolding cyclic amplification; RT-QuIC: real time quaking-induced conversion; * denotes that the structure of amplified products arising from recombinant PrP in RT-QuIC may be different than that produced by PMCA, potentially explaining the loss of infectivity seen with RT-QuIC.

The initial discovery of the agents responsible for TSEs enabled the further development of diagnostic approaches beyond basic clinical and microscopic histopathological descriptions. The isolation of a misfolded cellular protein, found exclusively in the brains of TSE-infected animals and solely capable of inducing disease [36], permitted the development of an array of diagnostic assays dependent on the sensitivity and specificity of antibody-antigen interactions. These assays, including western blotting [23], immunohistochemistry [25], and enzyme immunoassay (EIA) [26], capable of distinguishing the normally folded cellular prion protein (PrP^C^) and the misfolded, infectious isoform (PrP^res^/PrP^Sc^), are still considered the “gold standard” diagnostic approaches for CWD and other prion diseases (Figure 2).

The primary characteristic of the misfolded prion protein, PrP^res^, which these assays take advantage of—its resistance to harsh conditions including acid treatment and enzymatic protease digestion, allowed for the reliable detection of infected individuals with high specificity. The amyloid plaques initially identified with routine histochemical staining in the CNS were found to be intensively immunoreactive to serum prepared from rabbits inoculated with hamster scrapie amyloid [25]. Brain homogenates from infected deer were also found to have protease-resistant remnants of immunoreactive prion amyloid when analyzed by SDS-PAGE and immune-dot blotting [23]. Although the presence of the protease-resistant core of the infectious prion protein is common to all prion diseases, its localization in the CNS and its immunoreactive banding pattern on western blot were found to help distinguish one prion agent from another [2]. The immunoreactive plaques observed in the CNS of deer with CWD are considered florid in nature, for example, whereas those found in cattle with bovine spongiform encephalopathy appear more granular. The western blotting pattern of CWD is a triplicate of di-, mono-, and unphosphorylated PrP^res^ protein bands 21–27 kD in size, with the diphosporylated band being the most intense [29]. The banding appearance of bovine spongiform encephalopathy PrP^res^, in contrast, reveals a triplicate ranging from 17–28 kD, with di- and monophosphorylated bands frequently of equal intensities [37].

From the advancements made with various immunoassays, more sensitive approaches to CWD diagnosis quickly evolved. Postmortem immunohistochemical studies of samples collected in the field and from experimental challenge studies have highlighted several target tissues as early harbingers of CWD infection, most importantly the dorsal motor nucleus of the vagus (DMNV) in the obex region of the brainstem and the medial retropharyngeal lymph nodes (RLN)—which are still considered the “gold standard” postmortem diagnostic tissues for regulatory diagnosis [38,39,40]. In deer, the RLN becomes positive before the DMNV, with rare exception [41], making it the most sensitive target tissue in this species. Elk, in contrast, may be DMNV positive without evidence of infection found in the RLN; as a result, both tissues should be examined in these animals [42]. In both species, progressive deposition of PrP^res^ in the DMNV and other regions of the brain has allowed diagnosticians to estimate the stage of infection through subjective scoring approaches [43,44,45,46]. Tonsilar tissue, interestingly, was one of the first tissues showing evidence of immunodeposition following exposure, and has been used experimentally to identify infected animals antemortem [39,47,48,49]. Later studies found that lymphoid tissue in the caudal rectum may also serve as a prognosticator for CNS infection, providing further opportunities for antemortem diagnosis [46,50].

Over the course of these diagnostic field and experimental studies, the growing geographical extent of the disease was examined [51,52,53,54,55], and evidence was uncovered in both deer and elk which showed that the host’s prion gene (*PRNP*) sequence may modulate susceptibility [56,57,58,59,60,61,62]. Animals with several alleles harboring coding mutations, including 225S → F in mule deer [60], 132M → L in elk [61], and 96G → S in white-tailed deer [62], were underrepresented among animals found to be infected, and were therefore thought to have a lower relative risk of infection compared to their wild-type counterparts. Later studies more clearly demonstrated that cervids with these alleles were susceptible, though may have a more protracted incubation period than those with wild-type alleles [57]. Collectively, these introductory studies allowed researchers to estimate the geographical boundaries of CWD-endemic areas and assemble a preliminary picture of disease pathogenesis in cervids with a range of genetic backgrounds.

As efforts to better characterize CWD pathogenesis, especially routes of transmission, continued, it became necessary to pursue alternate strategies for prion detection in those biological samples thought to be involved. Immunohistochemistry was not a practical approach for bodily fluids and excreta like blood, saliva, urine, or feces. The presumably low levels of PrP^res^ in these samples also made identification difficult using conventional western blotting and EIA. The experimental exposure of susceptible species, then, became the most practical (albeit time consuming) mechanism for assessing infectivity in body fluids.

### 2.2. Bioassay

The early experiments characterizing the transmissibility of CWD, and later uncovering potential transmission routes, required an extensive reliance on both natural and experimental hosts. Initial studies in natural hosts—mule deer and elk [39,63,64]—were used to demonstrate that animal to animal contact and environmental contamination played very important roles in disease transmission. More granular studies in white-tailed deer, addressing the roles of specific bodily fluids and cellular components, soon showed that saliva and blood carried high levels of infectivity [20]; the roles of urine and feces at that time were less clear. Not long after, the development of transgenic murine models, susceptible to CWD, allowed for a more thorough examination of body fluids, greater consistency within and across experiments, and even permitted the titration of infectivity [21,65]. Transgenic mice helped further illuminate the role of specific blood fractions [66], and offered greater sensitivity in identifying infectivity in both feces and urine [19], as well as in the tissues of animals inoculated with these and other biological samples through secondary passage experiments [67]. While still widely used today, biological models for diagnostic purposes are extremely impractical for obvious reasons, including ethical considerations, costs, and prolonged incubation periods.

The development of antibody-antigen dependent assays (western blotting, IHC, and EIA) allowed for a better understanding of the pathogenesis of CWD and other prion diseases, and helped to identify the most appropriate tissues to collect and evaluate postmortem. As a result, the above described conventional testing strategies have helped elucidate the ever-growing range of CWD in North America and beyond, and have been used to identify cervid hosts with varying levels of susceptibility linked to the *PRNP* gene. Bioassays, meanwhile, have helped uncover the likely routes of transmission in bodily fluids, especially saliva—which could prove useful in developing antemortem tests. However, additional in vitro approaches, which could mimic the misfolding process that occurs in vivo, would need to be developed to allow for a more sensitive detection of infectivity in body fluids and other diagnostically appropriate samples.

## 3. The Present State of CWD Diagnostics

With the pioneering work of immunological and bioassay studies, much has been learned about the pathogenesis, transmission, and, equally important, the geographic distribution of CWD and other prion diseases. Although immunological tests were very *specific* for prion infection, concerns arose early on that these assays were not *sensitive* enough—suspicions often supported by bioassay findings [26,67,68]. Indeed, it is common practice to report CWD test results as “Not Detected”, instead of “Negative”, to acknowledge the so far unmeasured insensitivity of IHC, western blotting, and EIA. Because of ethical, practical, and monetary considerations, attention was turned from bioassay to other methods which might allow more rapid, sensitive, and cost-effective detection of CWD and other prion infections in vitro, using techniques and approaches common to the diagnosis of other infectious agents—including cell culture and various amplification techniques.

Concurrent with the development of more sensitive techniques for identifying CWD infected cervids, efforts have been made to shift the diagnostic focus in deer and elk from postmortem to antemortem detection. With the frequent movement of farmed and wild cervids and their byproducts across North America and beyond, it is becoming increasingly important to develop screening programs to prevent the introduction of CWD into new areas. Currently, farmed cervid herds in both the United States and Canada may enroll in voluntary herd health programs which facilitate the interstate or interprovincial sale of animals [69,70]. These programs typically require meticulous inventories and a consistent postmortem testing history and are commonly more stringent than the limitations placed on wildlife relocations—however they are not fail safe. In both farmed and wild cervids, antemortem testing prior to animal movement may add another layer of security to prevent the spread of CWD.

**Figure 3:**
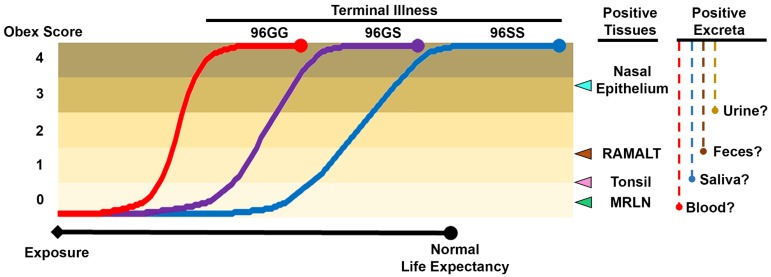
Model of the pathogenesis of chronic wasting disease in white-tailed deer with different *PRNP* backgrounds, with special attention on the diagnostic sensitivity of peripheral tissues and excreta. The disease seems to progress at different rates in animals with differing *PRNP* sequences, which affects the time points at which peripheral tissues may become positive by conventional or experimental diagnostic assays. Shedding in excreta is less well characterized, and the kinetics of prionemia, prionsialia, prionochezia, and prionuria (infectious prions in the blood, saliva, feces, and urine, respectively) may fluctuate during the course of infection. White-tailed deer with 96GG, GS, and SS *PRNP* sequences are considered, though the model would similarly apply to deer and elk with other variants of the *PRNP* gene. Obex scoring is a subjective, semi-quantitative method for visually estimating the amount of PrP^res^ deposition in the obex using immunohistochemistry, and has been used in studies in both deer and elk [44,45,46]. Data presented here have been compiled from several studies [19,39,42,43,44,45,46,47,48,49,71,72,73,74]. RAMALT: recto-anal mucosa associated lymphoid tissues; MRLN: medial retropharyngeal lymph node.

While progress has been made on assay development and antemortem testing strategies, some limitations remain. Bodily fluids have been shown to be infectious, and could therefore be used as a diagnostic sample—but little is known about the kinetics of shedding in bodily fluids over the course of disease. Easily accessible peripheral tissues (e.g., tonsil) have high diagnostic sensitivity late in the course of disease, but fall short when animals are in earlier stages. Lastly, a specific host genetic background, which has been linked to reduced susceptibility and/or delayed disease progression, may complicate detection in either bodily fluids or peripheral tissues (Figure 3). With a better understanding of CWD pathogenesis in all susceptible species and genetic backgrounds, the gains that have been made in sampling and testing approaches can more effectively be applied to improve both test sensitivity and specificity.

### 3.1. Amplification Assays for the Detection of Ultra-Low Levels of CWD Prions

Of the in vitro assays currently in development for detection of CWD prions, amplification assays are by far the ones getting the most attention [28,31]. At their very basic level, these assays take advantage of the proclivity of PrP^Sc^ to induce a conformational change in a normal cellular prion protein substrate (PrP^C^). They may make use of the high levels of PrP^C^ found in the brains of transgenic mice, for example, or they can rely on bacterial expression systems to produce large amounts of recombinant PrP^C^ for use as a conversion substrate. Amyloid fibril disruption and generation of new prion “seeds” for amplification may be accomplished by simple shaking or through sonication. The readouts of the assays may require blotting techniques to visually detect amplified aggregates of PrP^res^, paralleling conventional gel-based PCR, or they may take advantage of fluorescent molecules which bind to growing amyloid fibers, allowing a readout similar to real-time, quantitative PCR. In each case, the objective of these techniques is to amplify low levels of misfolded proteins in vitro which may be present in a sample, to levels which can be readily observed by more traditional methods (Figure 2).

### 3.2. Protein Misfolding Cyclic Amplification

The first of these amplification techniques to be adapted for use with CWD, which helped to lay the groundwork for future developments in CWD amplification-based diagnostics, was the protein misfolding cyclic amplification assay (PMCA) [29,67]. This assay requires, most importantly, a cellular prion protein substrate derived from brain homogenates of susceptible, or potentially susceptible, hosts. For detection of CWD, very often these homogenates are derived from transgenic cervidized mice, which may express high levels of white-tailed deer or elk PrP^C^, providing an abundance of substrate for in vitro conversion. The brains are commonly homogenized in phosphate buffered saline with a range of protease inhibitors and surfactants, to which the CWD-harboring sample, or “seed”, is added and allowed to incubate at 37 °C for 24–48 h. The samples are sonicated intermittently to fragment the growing amyloid chain. These new amyloid fragments may then serve as seeds for further conversion reactions. After each experiment, the seed-substrate preparations may be treated with protease and evaluated by western blot for the resistant conformer, or they may be passaged into a new preparation of brain homogenate, in the case of “serial” PMCA (sPMCA) [73,75]. Serial PMCA, not unlike nested PCR, may involve up to ten passages or more of amplification over the course of several weeks in an attempt to achieve even greater sensitivity than conventional PMCA.

Several modifications have been described which improve the sensitivity of PMCA or sPMCA, including the addition of plastic beads or putative cofactors [76,77]. Some researchers have essentially hybridized PMCA with the quaking induced conversion assay described below, and applied an electrical current in an effort to improve sensitivity [78]. To detect the misfolded protein, many permutations still rely on protease treatment which destroys the normal cellular protein, and potentially some protease-sensitive isoforms of the infectious proteins, ultimately reducing sensitivity. To circumvent protease treatment, one group reported using a surround optical fiber immunoassay (SOFIA) to specifically identify the disease-associated form of the prion protein using immunocapture in combination with laser-induced fluorescence [79,80]. Each of these modified approaches have shown potential for the detection of exquisitely low levels of CWD prions, perhaps down to the attagram level—potentially at the cost of reduced specificity as is commonly seen in other diagnostics using extended PCR or nested PCR protocols [77].

Variations of the PMCA assay have been used to explore various areas of CWD pathogenesis, e.g., to assess the potential for infectivity in body fluids and other excreta [19,77,81,82], and to detect low levels of misfolded protein in soil [83], water [84], and plant samples [85]. Notably, the CWD seeds generated in vitro by sPMCA have proven infectious to some degree in susceptible hosts [86], indicating that the technique may accurately model what occurs in vivo; therein lays its advantage among amplification strategies. Neither PMCA nor any of its derivatives have, however, been used extensively in field studies which would allow researchers to test the true sensitivity and specificity against conventional IHC or EIA. Ultimately, four important considerations continued to drive the development of new diagnostic amplification techniques beyond PMCA: (1) the ethical concerns raised by continued use of animal hosts for a PrP^C^ conversion substrate; (2) the need for an assay which could detect all potential infectious conformers of the prion protein, including protease-sensitive forms; (3) time constraints commonly required in field surveillance; and (4) the need for a technically simple assay with a practical read-out, one which could more easily allow for quantification.

### 3.3. Quaking Induced Conversion

Many of the considerations described above would be met by a conceptually similar technique developed nearly in parallel—the quaking induced conversion assay, or QuIC [31,87]. Importantly, this approach makes use of recombinant PrP^C^, an approach which has two distinct advantages over traditional PMCA: first, the protein substrate can be quickly and consistently produced in any cellular expression system, commonly *E. coli*, and second, it allows for the rapid design of substrates tailored to the researcher’s needs, without the complicated intermediate steps needed to generate transgenic mice. Commonly, a truncated form of the Syrian hamster PrP protein is used as a substrate, however a number of cervid and non-cervid recombinant substrates have been developed for the detection of CWD and other prions of both animals and humans [88,89].

The QuIC technique seemingly went unnoticed by those researching CWD, until modifications, including the incorporation of a fluorescent dye and a plate reader capable of stringent shaking protocols, allowed it to evolve into a format that satisfied each of the considerations which had hindered the widespread implementation of PMCA [30]. As with PMCA, the shaking is believed to disrupt growing amyloid fibrils and multiply the number of seeds available for further amyloid formation. The fluorescent dye, commonly thioflavin T, is thought to intercalate within the growing amyloid fibril. When bound to amyloid, thioflavin T exhibits a different emission spectrum than when free in solution, permitting the user to monitor amyloid amplification in real time. Like qPCR, this consolidates the assay read-out into a technically simple, unambiguous amplification curve which may additionally allow some level of quantification.

Current permutations of the real time QuIC (RT-QuIC) assay monitor changes in fluorescence every 15–60 min, over periods of time ranging from 24–96 h or more. As with sPMCA, longer RT-QuIC protocols allow for amplification of lower levels of misfolded prions, while concurrently risking spontaneous misfolding and decreased specificity. Under these different protocols, RT-QuIC has been used to examine the initial steps of CWD tissue invasion [74], quantify the levels of misfolded protein in bodily fluids [71], and evaluate inter- and intra-species susceptibility to CWD in vitro [89]. It has also been blindly evaluated in parallel with PMCA, IHC, and EIA [32,44,45,77], allowing a direct comparison between RT-QuIC and conventional diagnostic approaches. Generally, these studies have shown RT-QuIC is at least as sensitive as IHC or EIA.

The strengths of RT-QuIC lie in its consistency, malleability, rapidity and ease of interpretation. Because it relies solely on recombinant PrP^C^ as a conversion substrate, it is less capable of modeling the in vivo conversion process than PMCA. Importantly, the amplified products generated by RT-QuIC have not yet been shown to be infectious in vivo, as they have with PMCA. In fact, very few diagnostic approaches, short of viral or bacterial culture and isolation methods, are dependent on infectivity. Thus, neither of these caveats should prevent the implementation of RT-QuIC as a diagnostic approach for CWD or other prion diseases.

### 3.4. Tyramide Signal Amplification

While the goal of both PMCA and RT-QuIC is to amplify low levels of misfolded prions by seeded conversion, tyramide signal amplification instead magnifies the signaling mechanisms present in conventional assays, and has been used experimentally for CWD specifically to improve IHC detection in fixed tissues [74,90]. In the case of IHC, horseradish peroxidase-labeled antibodies bound to CWD prion antigen in situ activates the tyramide substrate which then accumulates in the immediate vicinity of the antibody, amplifying signal intensity up to 15-fold [91]. This technique has been used to more effectively track the early pathogenesis of experimental CWD in both transgenic mice and deer, though has not yet found its way into clinical applications.

### 3.5. Cervid Prion Cell Assay

Just as cell culture systems have been developed for the detection and diagnosis of a range of viruses and intracellular bacteria, cell lines have likewise been developed for the cultivation and quantification of infectious prions [27]. Researchers have inserted a variety of alternate PrP gene sequences into the mutable rabbit kidney epithelial RK13 cell line, which have rendered them susceptible to species-specific prion replication. In the cervid prion cell assay, or CPCA, expression of the elk PrP gene resulted in an RK13 line susceptible to CWD which permits the titration of an infectious dose comparable to bioassay in transgenic mice. Although the CPCA effectively decreased the time and cost required for bioassay, and models in vivo infection more closely than amplification assays, the culture of prions in susceptible cell lines still remains limited in practicality compared to PMCA and RT-QuIC. As viral isolation and bacterial cell culture remain staples of microbiological testing today, so may cell culture have a future in the diagnosis of CWD in cervids.

### 3.6. Sample Selection for Antemortem Testing

Past and present detection strategies have supported the work on CWD pathogenesis and demonstrated the kinetics of shedding in bodily fluids and excreta. Using amplification approaches, evidence of CWD prion presence has been reported in a range of bodily fluids [19,77,81,92,93,94], making them tempting targets for the development of novel diagnostic strategies. Studies in other model systems, including sheep and humans, have identified peripheral tissues which may also serve as a useful diagnostic sample and indicator of central nervous system infection [95,96,97]. Through these discoveries, antemortem testing for CWD is becoming increasingly more sensitive and reliable, and may someday prove useful for screening prior to animal movement (Table 1).

### 3.7. Bodily Fluids and Excreta

With many infectious diseases of veterinary and human importance, assays which utilize bodily fluids—especially blood—are considered ideal choices for a diagnostic test. CWD is not unique in this regard, and the primary focus has been on the development of a hematologic test to identify infected animals [93,94]. Very little is known about the kinetics of prionemia (prion infectivity in the blood), or the kinetics of prion shedding in other forms of excreta, and yet a number of primarily amplification-based studies have attempted to identify the misfolded protein in these samples. In many cases, these techniques have been developed using a very limited number of infected animals, and more importantly a limited number of negative controls [98,99]. Very rarely have the techniques been successfully applied to large field studies, although several laboratories continue to pursue testing of saliva and urine [71], blood [94], and fecal samples [72] collected from experimentally exposed animals or during depopulations of CWD-infected farmed deer and elk. These studies will eventually allow for more direct comparisons to be made with conventional postmortem testing and allow researchers to evaluate their sensitivity and specificity.

### 3.8. Accessible Peripheral Tissues

Several accessible tissues, including peripheral lymphoid and neuroepithelial tissues, have been identified which may help identify CWD-infected deer and elk antemortem or postmortem [44,45,46,100,101,102]. Each of these tissues offer both strengths and weaknesses in their diagnostic feasibilities, and need to be considered on a case by case basis in their application. For example, lymphoid tissues like tonsil—where CWD prions may accumulate early in the course of infection—have been found to be quite sensitive when compared to central lymphoid and nervous tissues collected postmortem. To that end, the direct sampling of medial retropharyngeal lymph nodes might be expected to offer near perfect sensitivity. The aforementioned tissues are, however, rather difficult to sample practically and repeatedly when compared to other, less sensitive peripheral tissues like recto-anal mucosal associated lymphoid tissue (RAMALT) [41]. Real-time QuIC analysis of olfactory neuroepithelial swabs, a relatively simple technique shown to be quite sensitive in the diagnosis of clinical Creutzfeldt-Jakob disease in humans, may only be effective in identifying deer and elk in the most terminal stages of CWD [44,45]. Accordingly, it should be remembered that irrespective of the sampling tissue and technique, or assay used, cases in the very early stages of infection may still test negative—making serial sampling indispensable for antemortem diagnosis. As more is learned about CWD pathogenesis and transmission, however, improvements in both tissue and body fluid sampling strategies will most certainly be made.

### 3.9. Sample Collection in Farmed and Free-Ranging Cervids

While post-mortem samples are relatively easy to collect on the necropsy floor or in the field, weather and equipment permitting, antemortem sampling presents its own unique challenges in both farmed and wild deer and elk. Farmed cervids are commonly collected in small groups, processed in modern handling systems and restrained in standard large animal squeeze chutes, which greatly facilitates the collection of accessible bodily fluid samples and rectal biopsies, for example [44,45,72]. More invasive biopsy collections from farmed cervids, including tonsil and retropharyngeal lymph node, requires deep sedation and anesthesia—a practice that is all but necessary for the collection of any samples from wild cervids [48,102]. Apart from the need for sedation or anesthesia, a more important factor limiting the efficiency at which wild cervids may be sampled is first finding and then capturing animals. Sampling in either group is not without risk, however, with farmed cervids occasionally suffering from severe injuries like broken limbs, and wild cervids at risk for the development of capture myopathy, an infrequent and often fatal syndrome resulting from the handling of wild cervids [103].

### 3.10. Genetic Background and Antemortem CWD Diagnostic Sensitivity

With the development of antemortem testing approaches came the discovery that an animal’s genetic background could have a profound effect on antemortem diagnostic sensitivity. In both deer and elk, RAMALT and nasal brush testing in animals with the prototypical *PRNP* genotype (96GG in deer and 132MM in elk) have been found to have the highest diagnostic sensitivity [44,45,46]. Antemortem testing in animals with a *PRNP* genetic background considered less susceptible, for example, 96GS or SS in deer and 132ML or LL in elk, is significantly less sensitive. Taken together with the apparently reduced susceptibility in animals with specific *PRNP* sequences, the reduced sensitivity of peripheral tissues can be best explained by a slower disease progression in these genotypes. In support of this, less susceptible animals which were CWD-negative in peripheral tissues were more commonly found to be in earlier stages of the disease, implying that the appearance of detectable prions in these peripheral tissues may be dependent primarily on disease stage, and not genetic background [44,45,46]. Several unanswered questions remain, however: do sensitivity limitations apply to all peripheral tissues? Do they apply broadly to all diagnostic assays? How might bodily fluids be affected? Should we use this information to encourage cervid farmers to breed highly susceptible animals to afford regulators a greater test sensitivity, or should we encourage a shift towards more resistant animals to help slow or prevent the spread of CWD? Ongoing research and policy discussions will hopefully provide the answers needed to move forward.

As with well-described bacterial and viral diagnostic strategies, diagnostic approaches for CWD and other TSEs began with clinical and postmortem pathologic detection methods. These strategies quickly progressed to more sensitive molecular approaches, which sought to identify the agent using amplification techniques, and shifted the focus from postmortem to antemortem diagnosis. While not perfectly sensitive compared to postmortem testing, currently deployed amplification techniques for CWD have a comparable sensitivity to assays for other important diseases—most notably bovine tuberculosis in cervids, although the ramifications of CWD misdiagnosis may be far more consequential. The available prion amplification approaches importantly take advantage of the infectious prion’s mechanisms for storing and reproducing information, just as PCR targets RNA and DNA molecules. In the case of TSEs, the ability to store and reproduce this information is imprinted in the structure of the abnormal prion protein itself. What other information may lay in this structure? Perhaps information which may encode strain, virulence, or zoonotic capacity? Can we identify a roadmap for pathogenesis or transmission in hosts with diverse genetic backgrounds? These are questions with absent or incomplete information, though with luck the tools currently in development will someday provide sufficient answers.

## 4. The Future of CWD Diagnostics

Detection capabilities for CWD and other infectious prions have progressed significantly over the past two decades, although there are still a number of areas requiring further research. First, demonstrating the improved sensitivity of prion amplification tests compared to conventional diagnostics is challenging, and will require well-structured experiments and well-defined samples in order for them to supplant immunohistochemistry or EIA. Second, there are critical gaps in epidemiologic studies which make it difficult to identify the source(s) of CWD introduced into previously naïve populations and to estimate environmental contamination in non-endemic areas. Finally, it should be remembered that while it is important to continue improving CWD diagnostics, it is equally important to translate these findings for the benefit human medicine, in the form of improved diagnostics for human prion diseases, Alzheimer’s disease and other protein misfolding disorders.

### 4.1. Improving Current CWD Diagnostic Approaches

As the pathogenesis of CWD is further defined in susceptible species and genetic backgrounds, improvements in sampling strategies should be expected. For example, lymph node aspirates and oral swabs, which are commonly used to diagnose a range of diseases in veterinary and human medicine [104,105,106], could be suitable for early antemortem diagnosis when combined with the appropriate testing platform. Fecal samples collected in the field, in contrast, may allow for a more passive sampling strategy to identify populations with otherwise undetectably low levels of CWD prevalence. Effective tissue and body fluid sampling has developed slowly over the past few decades, and there is every expectation that it will continue to evolve into the future.

Testing strategies are likewise evolving, with ever increasing sensitivity being reported by PMCA and RT-QuIC and other novel diagnostic approaches currently in development. A perpetual hurdle to demonstrating advanced sensitivity is the difficulty in overcoming a “gold standard”—how should we interpret samples which are positive by an amplification assay like sPMCA or RT-QuIC, yet IHC or EIA negative? To illustrate this point, experimental longitudinal studies have shown that sPMCA can identify misfolded prions in the blood of transgenic mice with a known exposure history [99]; however, the amplification experiments were not performed blindly, and thus should be interpreted with caution. Other, appropriately blinded studies have shown that both sPMCA and RT-QuIC more readily identify CWD prions in terminal cervid samples compared when to IHC—however, because of their terminal nature, it is very difficult to prove these animals were truly infected without a confirmatory test like bioassay to support the diagnosis [32,67]. Ideally, studies seeking to demonstrate the enhanced sensitivity of prion amplification approaches should *prospectively* incorporate both a longitudinal and blinded strategy, with repeated sampling of animals with a known and unknown exposure history to demonstrate presence or absence of infection, verified by IHC or EIA, in animals initially diagnosed by experimental techniques. Appropriate negative controls, including tissue or bodily fluid controls from negative hosts, are critical, while inter-lab validation is also an important strategy to consider, especially when a limited number of samples are under evaluation. It remains to be seen how well the quantitative or semi-quantitative nature of assays like RT-QuIC may correlate to in vivo infectivity: at what point does amplification-based detection become biologically relevant? Experiments such as these are ongoing, and may soon provide insight into the true sensitivity and specificity of prion amplification assays, and, perhaps as importantly, the true sensitivity and specificity of conventional and “gold standard” diagnostic approaches.

### 4.2. Exploring New Frontiers in CWD Diagnostics

Along with ongoing improvements in current sampling and testing strategies, future efforts should continue to pursue new and uncharted areas in CWD diagnostic capabilities. Several studies have demonstrated the occurrence of a number of putative CWD strains circulating in the wild [107,108], and while strain-typing is commonplace for viral or bacterial agents, no currently available approach has been shown to allow for rapid discrimination of diverse CWD prion strains. Western blotting very crudely identifies differences between CWD and BSE, while clinical presentation helps to differentiate sporadic CJD from variant CJD, for example, but current diagnostic technologies do not confer the ability to differentiate CWD strains or specifically identify the sources of new CWD incursions. The amplification-based assays could most likely address this diagnostic gap, with preliminary research seemingly demonstrating that RT-QuIC could provide reliable information regarding human Creutzfeldt-Jakob disease isolates [109]. This technology may effectively translate to CWD strains, where the comparison of various amplification parameters of cervid isolates in different amplification substrates could be employed. The ability to differentiate CWD isolates would be extremely helpful in epidemiologic studies, by allowing apparently new epidemic foci to be traced to specific geographic locations or source herds. Perhaps new strains would be discovered, including isolates previously undetectable by currently available technologies. Strain discrimination and characterization would additionally provide evidence and insight into prion evolution and adaptation—critical information which could be incorporated into field studies and efforts to investigate host resistance, and possibly help predict vaccine utility.

With the quantitative abilities of RT-QuIC, approximate titration of prion burden in biologic or environmental samples may also be possible using CWD amplification assays [71]. Early studies of saliva and other body fluids have shown variable levels of prion seeding potential in samples collected at different time points during infection, and it may soon be possible to correlate levels of shedding to incubation periods and genetic background as well as secondary underlying disease—renal dysfunction or perhaps even viral or bacterial co-infections, for example. An understanding of prion burden in tissues may provide a more thorough understanding of CWD pathogenesis and disease staging, and permit diagnosticians to select more appropriate ante- or post-mortem tissues for sensitive diagnoses. The ability to assess environmental contamination will allow wildlife biologists to monitor disease movement more easily, while simultaneously affording estimates of reduced infectivity following environmental decontamination efforts.

Advancements in CWD testing will certainly benefit from the introduction of prion amplification assays into the diagnostic repertoire. Multi-dimensional assays like RT-QuIC, which provides a range of information including amplification rate and efficiency in mutable substrates, seem poised to shed light on CWD strains and biological or environmental burdens which will allow for more detailed studies into disease epidemiology and pathogenesis. The benefits that this work provides will not be limited to cervid health, however.

### 4.3. Realms beyond CWD Diagnosis

The TSEs are increasingly regarded as models for other protein misfolding disorders of the CNS and other organ systems, including Alzheimer’s disease, Parkinson’s disease, and chronic traumatic encephalopathy (CTE) [110,111,112,113]. The application of the lessons learned through the course of investigations into CWD and other TSEs to the diagnostic challenges presented by these increasingly common human neurologic disorders should also be considered. The fundamental mechanisms directing the propagation of prions are not unlike those responsible for the accumulation of Alzheimer’s Aβ protein, or α-synuclein in Parkinson’s disease, and the techniques introduced by sPMCA or RT-QuIC should be transferrable with modifications to substrate and reaction conditions [114]. Efforts are currently underway to assess this potential, with promising results in both tau- and synucleinopathies.

Among TSEs, CWD perhaps represents the ideal model system for developing and deploying these prion amplification tests, in that it uniquely represents a proteinopathy affecting natural populations and is the only TSE currently expanding in distribution. Sample selection will undoubtedly vary between distinct proteinopathies and host populations; however a structured implementation of amplification assays for CWD would certainly help lay the groundwork for advancements in naturally occurring protein misfolding disorders in humans.

The future of CWD diagnostics depends on continued progress in the understanding of disease pathogenesis and the identification of suitable antemortem samples, and most importantly refinement and implementation of amplification assays like RT-QuIC. The potential for these assays to discriminate CWD strains and quantify tissue and body fluid burden will provide invaluable information for both epidemiologic studies and risk assessments. Challenges in the diagnosis of naturally occurring human proteinopathies will be offset by opportunities to implement CWD diagnostic strategies, making the continued development of these assays essential for advancements in human health.

## 5. Conclusions

Chronic wasting disease, a prion disease of deer and elk first reported five decades ago, now represents the last of the TSEs for which transmission and dissemination remain unchecked. The tools available to diagnosticians for identifying infected animals have steadily progressed over that timeframe from clinical and pathological descriptions to antibody-antigen dependent immunoassays, and more recently have begun incorporating qualitative and quantitative prion amplification techniques. These tools have provided a deep understanding of disease pathogenesis and transmission, and allowed animal health professionals to monitor the expanding geographical presence of CWD. Sampling techniques have likewise evolved, with shifts from postmortem to antemortem approaches targeting peripheral tissues and body fluids, and may someday offer the ability to screen animals prior to movement or selectively identify animals for removal. In the future, CWD diagnostics may also offer hope for the rapid discrimination of strains and assessment of tissue burden and environmental contamination. Although CWD’s role as the last remaining unmanaged TSE is a distinction neither agricultural nor wildlife professionals hold in high esteem, the discoveries over the past several decades have greatly assisted the continued development of assays directed toward protein misfolding disorders occurring in natural populations, and will ultimately benefit not just animal health but human health as well.

## Figures and Tables

**Table 1:** A summary of published diagnostic approaches for chronic wasting disease. Data sets from larger, comprehensive studies with postmortem data are included in the table. Other smaller or incomplete studies are referenced elsewhere in this review. Shaded rows indicate currently approved post-mortem diagnostic approaches for CWD. MRLN: medial retropharyngeal lymph node; RAMALT: recto-anal mucosa associated lymphatic tissue; CSF: cerebrospinal fluid; IHC: immunohistochemistry; EIA: enzyme immunoassay; RT-QuIC: real time quaking-induced conversion; sPMCA: serial protein misfolding cyclic amplification; NA: not applicable; ND: not determined.

	Sample	Method	Number Positive Postmortem (Total Examined)	Sensitivity *	Specificity *	Reference	Sample Notes
Tissues	Brainstem (obex)	IHC	NA	NA	NA	NA	IHC and ELISA of brainstem and RLN are considered the “gold standard” postmortem diagnostic approach for CWD. In deer, RLN are generally considered more sensitive, while in elk it is recommended both tissues be evaluated to confirm a diagnosis.
		EIA	53 (1986)	92%	100%	[26]	
	MRLN	IHC	NA	NA	NA	NA	
		EIA	84 (2042)	99%	>99%	[26]	
		RT-QuIC	23 (1243)	100%	100%	[32]	Field samples, postmortem
	Tonsil	IHC	100 (1150)	99%	100%	[49]	Field samples, postmortem
		sPMCA	30 (48)	ND ^†^	ND ^†^	[68]	Experimental animals, antemortem
	RAMALT	IHC	150 (561)	68%	>99%	[46]	Field samples, postmortem
		RT-QuIC	289 (409)	70%	94%	[45]	Field samples, antemortem
	Nasal brushings	RT-QuIC	289 (409)	16%	91%	[45]	Field samples, antemortem
Body Fluids/Excreta	Blood	RT-QuIC	16 (21)	93%	100%	[90]	Experimental animals, serial collection
	CSF	sPMCA	16 (37)	19%	100%	[79]	Field samples, postmortem
		RT-QuIC	26 (48)	50%	96%	[74]	Experimental animals, postmortem
	Saliva	RT-QuIC	18 (22)	78%	98%	[88]	Experimental animals, antemortem
	Urine	RT-QuIC	18 (22)	39%	100%	[88]	Experimental animals, antemortem
	Feces	sPMCA	5 (36)	ND ^†^	ND ^†^	[78]	Field samples, ante- and postmortem
		RT-QuIC	15 (25)	53%	100%	[97]	Field samples, antemortem

* The sensitivity and specificity of various approaches are compared to postmortem immunohistochemistry of the obex +/- retropharyngeal lymph nodes. ^†^ Sensitivity and specificity could not be calculated, since it was proposed that a number of samples in these studies were from CWD-positive animals which were IHC negative postmortem.
